# Model systems of protein-misfolding diseases reveal chaperone modifiers of proteotoxicity

**DOI:** 10.1242/dmm.024703

**Published:** 2016-08-01

**Authors:** Marc Brehme, Cindy Voisine

**Affiliations:** 1 Joint Research Center for Computational Biomedicine (JRC-COMBINE), RWTH Aachen University, 52062 Aachen, Germany; 2 Department of Biology, Northeastern Illinois University, Chicago, IL 60625, USA

**Keywords:** Chaperone, Co-chaperone, Chaperome, Proteostasis network, Protein-misfolding disease, Disease models

## Abstract

Chaperones and co-chaperones enable protein folding and degradation, safeguarding the proteome against proteotoxic stress. Chaperones display dynamic responses to exogenous and endogenous stressors and thus constitute a key component of the proteostasis network (PN), an intricately regulated network of quality control and repair pathways that cooperate to maintain cellular proteostasis. It has been hypothesized that aging leads to chronic stress on the proteome and that this could underlie many age-associated diseases such as neurodegeneration. Understanding the dynamics of chaperone function during aging and disease-related proteotoxic stress could reveal specific chaperone systems that fail to respond to protein misfolding. Through the use of suppressor and enhancer screens, key chaperones crucial for proteostasis maintenance have been identified in model organisms that express misfolded disease-related proteins. This review provides a literature-based analysis of these genetic studies and highlights prominent chaperone modifiers of proteotoxicity, which include the HSP70-HSP40 machine and small HSPs. Taken together, these studies in model systems can inform strategies for therapeutic regulation of chaperone functionality, to manage aging-related proteotoxic stress and to delay the onset of neurodegenerative diseases.

## Abstract (precis)

**Summary:** Brehme and Voisine perform a comprehensive literature survey that highlights the power of model systems to unveil key chaperone modifiers of proteotoxicity with potential therapeutic implications.

## Introduction

Chaperones and co-chaperones, collectively referred to as the chaperome (Box 1), work together in a network of macromolecular protein folding, refolding and degradation machines. These machines guide chaperone-dependent client proteins from the very beginning of ribosomal exit during translation of the nascent polypeptide chain to native folding at the cellular target location (Hartl et al., 2011), through to their eventual clearance when nonfunctional or present in excess (Schmidt and Finley, 2014). The chaperome consequently represents a highly complex and modular molecular system. Chaperome client proteins differ in abundance, structure and biochemical state depending on the cellular environment during development and aging, stress or homeostasis, in health and disease (Taipale et al., 2014). This diversity necessitates that the physical and functional interactions amongst chaperones and co-chaperones are highly plastic.

**Box 1. Glossary**

**Chaperome:** The interconnected network of chaperones and co-chaperones that work cooperatively in cellular protein folding, refolding and degradation.

**Chaperone**: Protein that facilitates the folding, refolding and assembly of macromolecular structures, either through the use of ATP in cycles of substrate binding and release, or in an ATP-independent ‘holding’ mode.

**Co-chaperone:** Protein that assists and modulates its cognate chaperone's activity.

**Proteostasis:** The combined action of processes by which cells maintain a ‘healthy’ proteome, including proper native folding, interaction profile, concentration, and subcellular localization.

**Proteome:** The ensemble of proteins expressed from protein-coding genes in the human genome by a particular cell at a given point in time, including splice variants and post-translationally modified protein species.

**Interactome:** The ensemble of molecular interactions in a particular cell at a given point in time, including physical protein-protein interactions.

**Foldases/folders:** Molecular chaperones that utilize ATP in cycles of substrate binding and release in order to support the folding or refolding of their cognate client proteins.

**Holdases/holders:** Molecular chaperones that act in an ATP-independent manner by binding and shielding exposed hydrophobic regions of nascent or misfolded polypeptides as well as folding intermediates to prevent their aggregation or aberrant interaction with cellular proteins.

The protein folding pathway is highly conserved throughout evolution, from bacteria to humans (Hartl and Hayer-Hartl, 2002; Hartl et al., 2011). The chaperome functions as a protective system to ensure proper folding of newly synthesized proteins and prevention of protein misfolding and aggregation. Chaperones bind to exposed hydrophobic regions of nascent or misfolded polypeptides, shielding these residues from forming aberrant interactions with cellular proteins. This ‘holding’ activity of a chaperone does not require ATP; however, protein folding and refolding requires an ATP-dependent cycle of substrate binding and release (Mayer and Bukau, 2005; Horwich et al., 2007; Liberek et al., 2008; Saibil, 2008). Various co-chaperones catalyze cycling of ATP-driven folding machines, along with providing client specificity (Caplan, 2003). Together, chaperone ‘folders’ and ‘holders’, along with co-chaperones, work in concert to ensure a properly folded proteome.

The chaperome consists of multiple functional families with a varying number of members, including the heat shock protein (HSP) families HSP100, HSP90, HSP70, HSP60; the chaperonins; HSP40 or DNAJ proteins; and the small HSPs (sHSPs), where names of each family correspond to the molecular weight of the original founding member (Hartl and Hayer-Hartl, 2002). In addition, there are organelle-specific chaperones of the endoplasmic reticulum (ER) (Kleizen and Braakman, 2004) and mitochondria (MITO) (Tatsuta et al., 2005), and tetratricopeptide repeat (TPR)-domain-containing co-chaperone family members (Hartl and Hayer-Hartl, 2002). A genome-wide survey of conserved functional domains defined by biochemical analyses identified over 300 genes encoding components of the human chaperome, represented by 88 chaperones (27%), of which 50 are ATP-dependent, and 244 co-chaperones (73%) (Brehme et al., 2014). As a highly conserved cellular quality control system and central arm within the proteostasis network (PN), defects in the human chaperome affect cellular proteome maintenance and repair capacity (Brehme et al., 2014). In line with this, chaperones and co-chaperones are implicated in an increasing number of protein-misfolding diseases (Kim et al., 2013; Kakkar et al., 2014).

Protein misfolding and aggregation have been associated with several age-related neurodegenerative diseases, and our understanding of the molecular underpinnings of these diseases is an ongoing area of scientific investigation. The increase in average human lifespan, along with disease demographic considerations such as a predicted three- to fourfold increase in Alzheimer's disease (AD) cases alone by 2050, foreshadows a significant socio-economic burden resulting from neurodegenerative diseases in the coming decades (Meek et al., 1998; Dobson, 2015). Many neurodegenerative diseases, such as AD, Parkinson's disease (PD), Huntington's disease (HD), and amyotrophic lateral sclerosis (ALS) are characterized by abnormal aggregation of misfolded proteins, i.e., amyloid β (Aβ) and tau in AD; α-synuclein in PD; huntingtin in HD, and TAR DNA-binding protein (TDP-43, also known as TARDBP) in ALS (Knowles et al., 2014). A growing body of evidence strongly suggests that failures or insufficiencies in various molecular chaperone systems are involved in the pathology of neurodegeneration.

Transcriptome analyses have been useful in identifying patterns of specific chaperones and co-chaperones that are upregulated or downregulated in response to cellular stressors, such as genetic, environmental or chemical perturbations, that challenge the folding of the proteome (Gasch et al., 2000; Lamitina et al., 2006). Organismal aging entails chronic stress on the proteome; thus, significant changes in chaperome expression could represent adaptive responses of a neuroprotective nature to an increasing proteotoxic burden or, conversely, could reflect the failure of chaperome function to protect the cellular proteome (Halaschek-Wiener et al., 2005; Gidalevitz et al., 2006). A systematic analysis of chaperone and co-chaperone gene expression dynamics during aging revealed that one third (32%) of chaperome genes were significantly repressed in human aging brains when compared with overall repression of genes in the aging genome (Brehme et al., 2014). Interestingly, this selective repression of the chaperome affects the major ATP-dependent chaperones and the full spectrum of chaperome families, whereas ATP-independent chaperones are mostly unaffected by repression, pointing towards a potential link with declining mitochondrial function in aging organisms (Lund et al., 2002; Zahn et al., 2006). The HSP40, HSP60 and HSP70 families were amongst the most repressed chaperones, with HSP70s being the most repressed group overall. However, in contrast with the broad spectrum of repressed chaperone families, sHSPs and the TPR co-chaperone proteins were the only families that were significantly induced. This selective activation of specific chaperome families could indicate that these proteins are involved in a general cellular stress response.

Aging correlates with cognitive decline and represents the most significant risk factor for dementia and neurodegenerative diseases (Brookmeyer et al., 1998; Hof and Morrison, 2004; de Lau and Breteler, 2006). Intriguingly, age-related changes in chaperome gene expression observed in aging brains are exacerbated in age-onset neurodegenerative diseases (Brehme et al., 2014). It is possible that differences in chaperome expression dynamics in individuals account for varying degrees of disease predisposition, age-of-onset, or severity of disease progression. Therefore, identifying the functionally decisive chaperone machineries that collapse and fail to prevent the overwhelming misfolding ‘crisis’ that occurs during normal aging could guide chaperome-directed therapeutic intervention of misfolding diseases.

Models of protein-misfolding diseases have been invaluable for establishing the contribution of the chaperome to proteostasis maintenance. Here, we survey the relevant literature covering the last 16 years and report on studies that utilized widely adopted model organisms expressing a subset of misfolding disease-related proteins. We outline directed candidate gene approaches that demonstrated a crucial role for major chaperome families in maintaining proteostasis. In addition, we use the collective power of diverse genetic studies to highlight the shared chaperome families identified in screens as modulators of proteotoxicity. Our quantitative analysis of the frequency with which specific chaperones and co-chaperones were identified reveals an overrepresentation of sHSPs and the HSP70-HSP40 machine. Guided by the compilation of data generated during this representative literature survey, we discuss potential concepts for therapeutic intervention strategies that target these key chaperones during human protein-misfolding diseases.

## Model systems of protein-misfolding diseases

Our survey of research articles using model systems of protein-misfolding diseases reaches back to the release of the human genome sequence in 2000. We selected representative studies that examined the effects of chaperones and co-chaperones in terms of enhancement or suppression of proteotoxicity associated with expression of disease-related proteins. Although there is a substantial wealth of studies using various model systems, our selection focused on the most commonly used genetically amenable model organisms – yeast, worms and flies – that express one of the following misfolded disease-associated proteins: a polyglutamine (polyQ) protein such as huntingtin, α-synuclein, Aβ, tau, superoxide dismutase 1 (SOD1), TAR DNA-binding protein (TDP-43), fused in sarcoma RNA-binding protein (FUS), or the Sup35 prion protein. It has been proposed that misfolding of these proteins places a chronic burden on the cellular folding network, which, over time, exceeds the proteostasis boundary, leading to proteostasis collapse (Gidalevitz et al., 2006; Powers et al., 2009; Olzscha et al., 2011; Morimoto, 2014). Genetic approaches using these disease models demonstrate that the chaperome plays a crucial role in protecting cells from proteotoxicity.

The functional role of chaperome families or individual family members can be investigated using a variety of genetic approaches guided by phenotypic readouts for changes in proteotoxicity. Research articles considered in this survey manipulate modifier genes either by overexpression (Warrick et al., 1999; Auluck et al., 2002; Krobitsch and Lindquist, 2000; Chan et al., 2000, 2002; Shulman and Feany, 2003; Cao et al., 2005; Al-Ramahi et al., 2006; Cooper et al., 2006; Tam et al., 2006; Bilen and Bonini, 2007; Kaltenbach et al., 2007; Liang et al., 2008; Sadlish et al., 2008; Roodveldt et al., 2009; Yeger-Lotem et al., 2009; Elden et al., 2010; Vos et al., 2010; Ju et al., 2011; Sun et al., 2011; Duennwald et al., 2012; Wolfe et al., 2013, 2014; Kim et al., 2014; Fonte et al., 2008), through knockdown (Fonte et al., 2002; Nollen et al., 2004; Kraemer et al., 2006; Kaltenbach et al., 2007; Hamamichi et al., 2008; Kuwahara et al., 2008; Van Ham et al., 2008; Roodveldt et al., 2009; Wang et al., 2009; Zhang et al., 2010; Calamini et al., 2011; Silva et al., 2011; Brehme et al., 2014; Khabirova et al., 2014; Jimenez-Sanchez et al., 2015), or by introduction of point mutations (Warrick et al., 1999; Chan et al., 2000, 2002) or deletions (Fernandez-Funez et al., 2000; Krobitsch and Lindquist, 2000; Willingham et al., 2003; Giorgini et al., 2005; Ritson et al., 2010; Butler et al., 2012) in order to explore their role in diseases of protein misfolding. Our survey covers different measures of proteotoxicity, evaluating the enhancement or suppression of various phenotypes as a readout for chaperone functionality, including aggregation (Krobitsch and Lindquist, 2000; Fonte et al., 2002; Nollen et al., 2004; Tam et al., 2006; Wang et al., 2007, 2009; Hamamichi et al., 2008; Sadlish et al., 2008; Van Ham et al., 2008; Roodveldt et al., 2009; Vos et al., 2010; Zhang et al., 2010; Calamini et al., 2011; Ju et al., 2011; Silva et al., 2011; Sun et al., 2011; Walter et al., 2011; Duennwald et al., 2012; Wolfe et al., 2013), neurodegeneration (Warrick et al., 1999; Chan et al., 2000, 2002; Fernandez-Funez et al., 2000; Kazemi-Esfarjani and Benzer, 2000; Auluck et al., 2002, 2005; Shulman and Feany, 2003; Al-Ramahi, 2006; Bilen and Bonini, 2007; Kaltenbach et al., 2007; Hamamichi et al., 2008; Wang et al., 2009; Ritson et al., 2010; Vos et al., 2010; Jimenez-Sanchez et al., 2015), or cellular toxicity (Willingham et al., 2003; Cao et al., 2005; Giorgini et al., 2005; Cooper et al., 2006; Kraemer et al., 2006; Kuwahara et al., 2008; Liang et al., 2008; Yeger-Lotem et al., 2009; Elden et al., 2010; Vos et al., 2010; Calamini et al., 2011; Ju et al., 2011; Sun et al., 2011; Butler et al., 2012; Wolfe et al., 2013, 2014; Brehme et al., 2014; Khabirova et al., 2014; Kim et al., 2014). Based on this collection of studies, we first review findings based on the use of a defined single candidate gene to demonstrate a role for each of the major chaperome families in modifying proteotoxicity. Next, we compile and report on those chaperones and co-chaperones that were identified in unbiased screens in various model systems (Table 1), grouped by respective misfolding diseases, listing the approach and phenotypic readout, and noting the human orthologs.

**Table 1.**
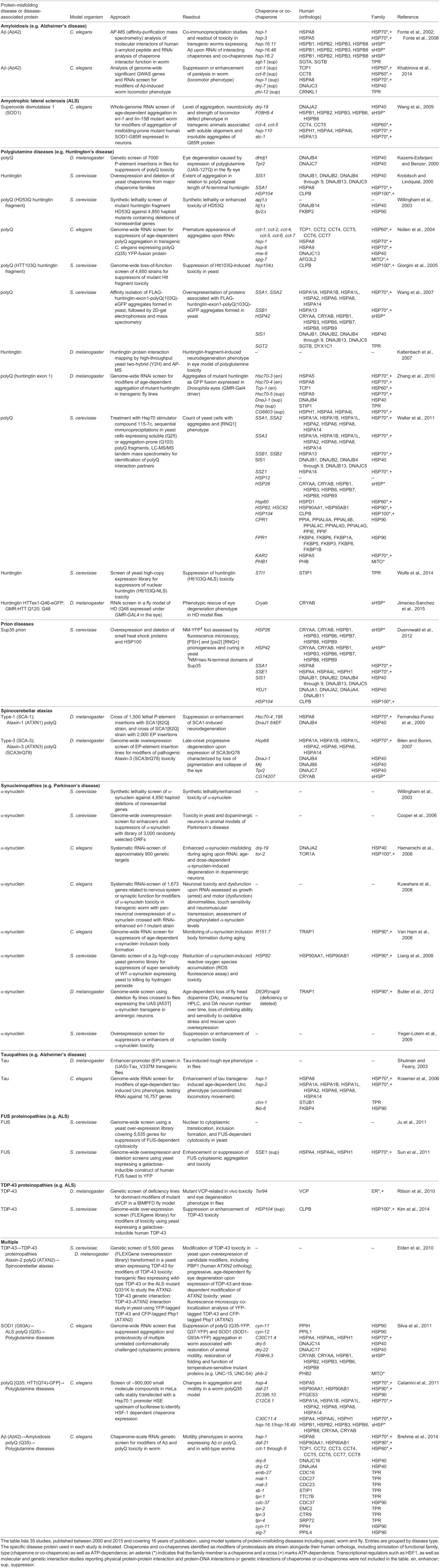
**Chaperones and co-chaperones identified in model-based studies of protein-misfolding diseases**

## Major chaperome families participate in proteostasis maintenance

Many studies based on model systems support a role for candidates from each of the major chaperome families; HSP100, HSP90, HSP70, HSP60, HSP40, sHSPs, and TPR-domain-containing proteins in proteostasis. These studies center on expressing disease-associated genes in model systems, establishing toxicity by monitoring neurodegeneration or growth, and testing whether changing the expression level of a specific chaperone or co-chaperone modifies toxicity. Subtle alterations in morphology of aggregates or aggregate number can also be monitored, providing additional measures for investigating the role of the chaperome in disease. Early studies demonstrated that overexpression of a specific human HSP70 (HSPA1L) in a *Drosophila* disease model suppressed neurodegeneration associated with expression of polyQ-containing forms of both ataxin 3 or androgen receptor, and α-synuclein (Warrick et al., 1999; Chan et al., 2000, 2002; Auluck et al., 2002). In a yeast model expressing the N-terminal fragment of a polyQ-containing huntingtin protein, overexpression of the yeast HSP70 (*SSA1*) reduced aggregate formation, whereas the dominant-negative version of the fly homolog to HSPA1L, *Hsc4-K71S,* enhanced neurodegeneration (Warrick et al., 1999; Chan et al., 2000, 2002; Krobitsch and Lindquist, 2000; Tam et al., 2006). These studies revealed that an increase in levels of HSP70 reduced aggregation of disease-associated proteins, thus playing a neuroprotective role.

Candidates from the remaining chaperome families have also been tested using a similar targeted approach. For example, overexpression of the human TPR domain-containing co-chaperone CHIP suppresses neurodegeneration in fly models expressing polyQ-containing versions of ataxin 1 and the N-terminal huntingtin fragment (Al-Ramahi et al., 2006). Likewise, overexpression of the yeast TPR-domain-containing co-chaperone *STI1* suppresses toxicity in a yeast model expressing the expanded huntingtin fragment (Wolfe et al., 2013). The co-chaperone HSP40 (*dHdj-1* and *SIS1*) and the nucleotide exchange factor *SSE1* that specifically modulate HSP70 activity were also shown to suppress toxicity and aggregation in yeast and fly disease models (Chan et al., 2000; Krobitsch and Lindquist, 2000; Sadlish et al., 2008). In addition to co-chaperones, overexpression of the human sHSP (HSPB7), a *Caenorhabditis* *elegans* HSP100 homolog (*tor-2*), and the yeast HSP60 subunit (*CCT-1*) and HSP90 homolog (*HSP82*) reduced toxicity and aggregation (Cao et al., 2005; Tam et al., 2006; Liang et al., 2008; Vos et al., 2010). Taken together, the directed examination of overexpression of candidates from the major chaperome families argues for the chaperome playing a crucial role in maintaining proteostasis and cellular health.

It is also worth noting that within certain families, specific members had greater protective effects compared with others. The cytosolic HSP60 CCT/TRiC complex contains eight subunits, yet only overexpression of subunits *CCT1* and *CCT4* reduced toxicity in yeast expressing the expanded huntingtin fragment (Tam et al., 2006). The remaining six subunits had little to no effect. Of the two HSP40 family members tested, *SIS1*, but not *YDJ1*, reduced aggregation in the yeast model expressing the huntingtin fragment (Krobitsch and Lindquist, 2000). Furthermore, the yeast HSP100 homolog *HSP104* did not suppress neurodegeneration in worms, whereas the overexpression of a *C. elegans* HSP100 *tor-2* restored neuronal health (Cao et al., 2005). Each of these directed studies in a model system for protein-misfolding disease suggest that certain chaperome families and specific members within these families play a more important role in proteostasis than others.

**Fig. 1.**
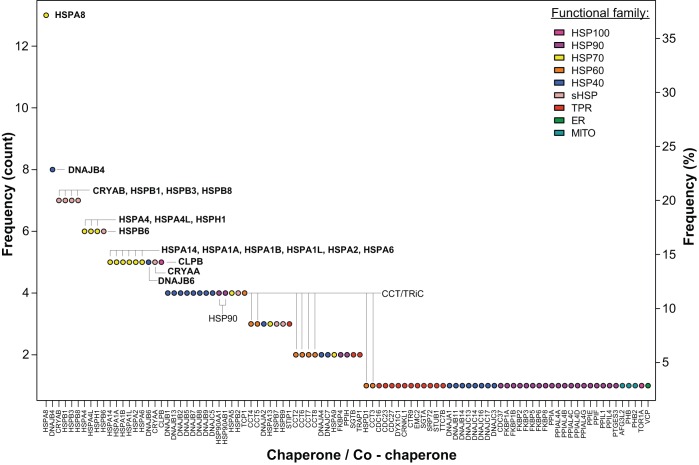
**Chaperones and co-chaperones identified in model-based studies of protein-misfolding diseases.** Frequency is indicated based on 258 overall occurrences of 95 unique chaperones and co-chaperones, corresponding to 35 studies examined (see Table 1), of which 27 studies identified chaperones and co-chaperones. Chaperones or co-chaperones identified five or more times are highlighted in bold. Functional chaperome family membership is annotated by color (see key). HSP40, HSP60, HSP70, HSP90 and HSP100 are heat shock protein families of molecular chaperones as defined by the molecular weight (40, 60, 70, 90 or 100 kDa, respectively) of the original founding member; sHSP, small heat shock protein; TPR, tetratricopeptide repeat domain-containing co-chaperone; ER, endoplasmic reticulum-specific chaperones and co-chaperones; MITO, mitochondria-specific chaperones and co-chaperones.

**Fig. 2.**
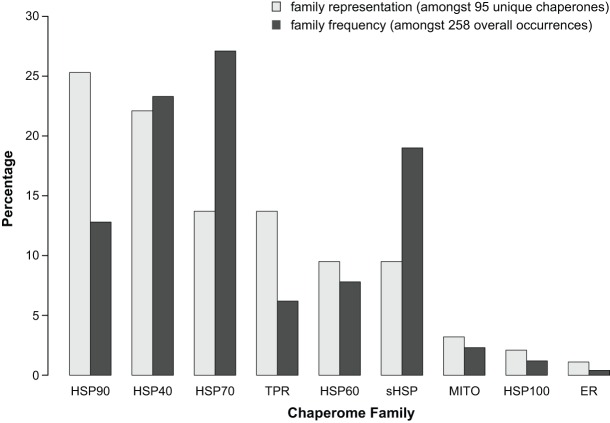
**Representation of chaperome functional families.** Percentage representation of individual chaperome families as defined in the legend of Fig. 1 amongst 95 uniquely identified chaperones and co-chaperones, and amongst 258 overall occurrences of all chaperones and co-chaperones in all studies highlighted in Table 1.

Studies using a candidate gene approach established a fundamental role for the chaperome in protein-misfolding diseases. Furthermore, these studies suggested a differential importance of specific chaperome families and individual members within families in proteostasis. However, most of these studies examine only one model system expressing one disease protein and measure a single readout, limiting our ability to interpret individual contributions of chaperome components to proteostasis. Over the last 16 years, experimental tools to manipulate the genome, including yeast deletion and overexpression libraries, and RNAi libraries in *C. elegans*, have emerged, allowing a less biased approach to uncovering the effect of chaperome families and their individual members in proteostasis. We selected a set of studies that attempt to be more comprehensive in their testing of chaperones, utilize genome-wide screens to provide an unbiased sampling of genes, or examine proteins associated with aggregates (Table 1). These studies employ different model systems, disease proteins, cell types and phenotypic readouts of proteotoxicity. Harnessing the collective power of these diverse genetic studies, we compiled a list of the chaperones and co-chaperones identified in each study and determined the frequency at which each individual candidate was found. We propose that a high frequency of identification reflects a consistent, vital role for specific chaperome families and members in proteostasis maintenance, overcoming the diversity of the experimental approaches and model systems. Of the 35 studies listed in Table 1, 27 studies identified 258 chaperone and co-chaperone occurrences in total, corresponding to 95 unique chaperones or co-chaperones (Figs 1 and 2). Below, we highlight trends revealed by our literature survey of protein-misfolding disease models, which implicate the HSP70-HSP40 machine and sHSPs as key chaperone modifiers of proteotoxicity.

## Model systems highlight a role for HSP70 in protein-misfolding diseases

**Fig. 3.**
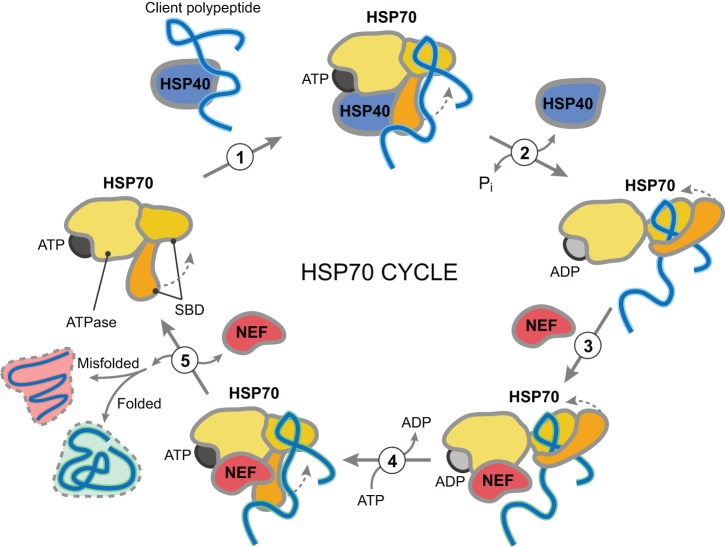
**HSP70 reaction cycle.** (1) HSP40 binds to a client polypeptide and interacts with HSP70. (2) The ATP-bound form of HSP70 interacts with the unfolded polypeptide via its substrate-binding domain (SBD) and upon the hydrolysis of ATP to ADP stimulated by HSP40, a more stable interaction between the ADP-bound form of HSP70 and the polypeptide is formed. (3) A nucleotide exchange factor (NEF) interacts with the HSP70:polypeptide complex and (4) allows the exchange of ADP for ATP. (5) Following the exchange of ADP for ATP, both the polypeptide and NEF are released from HSP70. If the polypeptide is not properly folded, it can enter another round of the HSP70 reaction cycle.

**Fig. 4.**
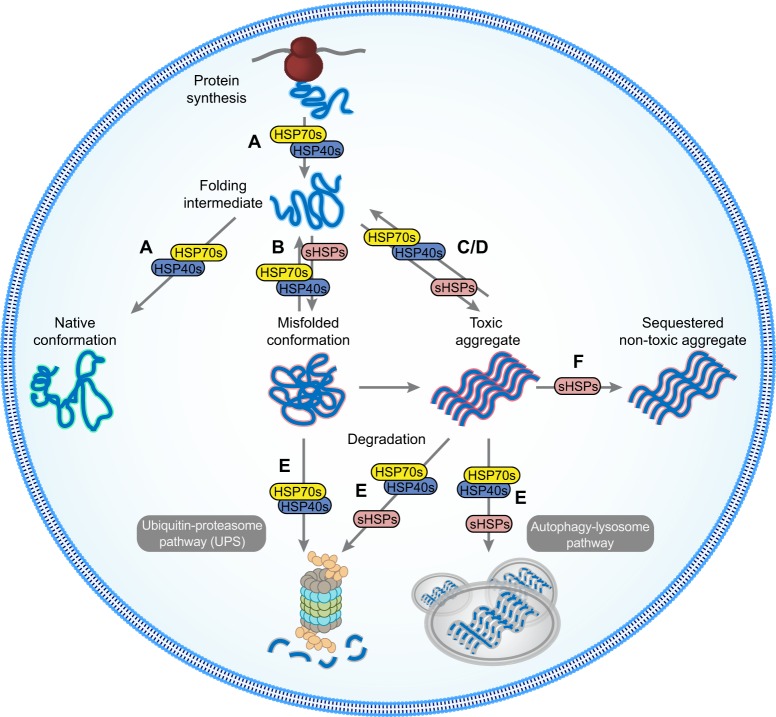
**Key chaperome modifier activities in misfolding-disease progression.** HSP70s and their HSP40 co-chaperones function in a variety of basic cellular quality control processes. Distinct combinations of HSP70s and HSP40s facilitate folding (A), refolding of misfolded proteins (B), preventing aggregation (C) or promoting disaggregation (D), and degradation of misfolded proteins (E). Recent therapeutic strategies have focused on partitioning HSP70 activity towards prevention of aggregation (C), disaggregation (D) and degradation (E) to maintain the integrity of the proteome. sHSPs also manage misfolded proteins (B-E) and also act as cellular shields, interacting with misfolded or aggregated proteins to prevent aberrant interaction with cellular proteins (F). In this capacity, sHSPs can interact with disease protein aggregates, sequestering these toxic aggregates and protecting cells.

Notably, HSP70s were identified most consistently across studies employing unbiased approaches (Table 1). Out of the 19 individual chaperome family members that were identified five or more times, 53% were HSP70s (Figs 1 and 2). HSP70s function in a variety of basic cellular quality control and maintenance processes, such as proper folding of newly synthesized proteins, along with preventing protein misfolding and aggregation through the binding of exposed hydrophobic residues. Iterating through cycles of client engagement and release coupled to ATP-binding and hydrolysis, HSP70s act to prevent aberrant protein-protein interactions. A simple model of the HSP70 reaction cycle asserts that the ATP-bound form of an HSP70 protein interacts with an unfolded protein (Fig. 3). Upon hydrolysis of ATP to ADP, a more stable interaction between the ADP-bound form of HSP70 and its substrate protein is formed. Following the exchange of ADP for ATP, the substrate protein is released from HSP70 (Mayer and Bukau, 2005; Mayer, 2013). Through this activity, HSP70s play a central role in all aspects of protein biogenesis and proteome maintenance, with effects ranging from stabilizing the native fold of an individual protein to contributing to proper protein interactome wiring at the systems level (Fig. 4).

There are at least 17 human HSP70 family members, including both constitutively expressed isoforms (HSC70) and stress-inducible HSP70s, of which 13 appear amongst all chaperones identified by our survey. The chaperone most frequently identified in our literature survey is the constitutively expressed cytoplasmic HSC70 chaperone, whose human ortholog is HSPA8 (Table 1, Fig. 1). 48% of the studies that found chaperones/co-chaperones (27 studies), or 37% of all studies considered (35 studies), identified this family member. The HSP70 family members observed at least five times or more are HSPA8 (13×), HSPA4 (6×), HSPA4L (6×), HSPH1 (6×), HSPA14 (5×), HSPA1A (5×), HSPA1B (5×), HSPA1L (5×), HSPA2 (5×) and HSPA6 (5×) (Fig. 1), where HSPA1A, HSPA1B and HSPA1L are all highly inducible upon stress. Based on our selection of studies, HSP70 family members influenced proteotoxicity in flies, worms and yeast expressing the disease proteins selected, except for TDP-43 and α-synuclein. Although HSP70 was not identified as a modifier of α-synuclein in the screen studies we selected, directed overexpression of HSP70 has been shown to reduce α-synuclein-related proteotoxicity, supporting a central role for HSP70 in diseases of protein misfolding (Auluck et al., 2002). While a genome-wide overexpression screen did not identify HSP70 as a suppressor or enhancer of TDP-43 associated toxicity in yeast (Kim et al., 2014), a closer examination of a single HSP70 may be of value as HSP70 physically interacts with TDP-43 aggregates (Udan-Johns et al., 2013). Furthermore, HSP70 family members were not identified in studies that utilized deletion libraries in yeast. This might be because deletion of one HSP70 family member could lead to upregulation of a different HSP70 family member, compensating for the loss of the candidate HSP70 gene. Using a combinatorial approach, i.e., by deleting multiple family members simultaneously, Meriin et al. (2002) provided support for the prominent role for HSP70 in proteostasis. To conclude, our analysis of the data gathered from a diverse set of genetic studies covering 16 years reveals the HSP70 family, and particularly HSPA8, as the centerpiece for maintaining cellular health in the presence of misfolded proteins linked to disease.

## Model systems uncover specific HSP40s (DNAJs) in protein-misfolding diseases

Of the overall 258 occurrences of chaperome family members identified in the studies reviewed in Table 1 (35 studies), HSP70s make up the largest fraction (27%) amongst all chaperome families. Interestingly, the next most frequently identified family is the HSP40s, at 23% (Fig. 2). HSP40s play a fundamental role as part of the HSP70-HSP40 system, as co-chaperones, stimulating HSP70 ATP hydrolysis (Fig. 3) (Kampinga and Craig, 2010; Kakkar et al., 2014). Based on the studies analyzed herein, changes in HSP40 functionality could compromise folding efficiency and HSP70 client specificity, putting cellular proteostasis at risk (Heldens et al., 2010). HSP40 family members do not exhibit the high level of conservation that is observed for HSP70s. However, all HSP40s possess a highly conserved 70 amino acid motif, termed the J domain, which interacts with HSP70s to stimulate ATPase activity (Kampinga and Craig, 2010). The remainder of the HSP40 protein sequence is of a more diverse nature, possibly contributing to and enabling heterogeneity and specialization of HSP40 function in specific aspects of the central role of HSP70s in protein biogenesis and proteome maintenance (Fig. 4). There are at least 48 human HSP40 family members, with the two most frequently implicated HSP40s being DNAJB4 (8×) and DNAJB6 (5×) (Fig. 1). The vast majority of the studies that identified DNAJB4 and DNAJB6 involved model systems expressing disease proteins containing polyQ expansions. Of the 11 studies using polyQ expansions, 45% identified DNAJB4 and 27% identified DNAJB6. Consequently, our survey pinpoints those two specific HSP40s out of 48 family members as the most relevant for polyQ expansion diseases, as judged by frequency of observations in a diverse range of studies, model systems and readouts.

Interestingly, human gene expression data supports the findings of genetic studies in small model systems. The HSP70 and HSP40 family members exhibit significantly altered expression dynamics during aging in the human brain, both being consistently repressed with age (Brehme et al., 2014). Among repressed genes, HSP40s were found to show significant changes as a family, with 62% of overall 48 HSP40 family members repressed in aging brain (superior frontal gyrus), 51% repressed in AD, and 41% repressed in both aging and AD. These changes in chaperome gene expression in aging and age-onset neurodegenerative disease highlight the value of using model systems to uncover important chaperone modifiers of proteotoxicity associated with human disease.

## Model systems reveal a role for sHSPs in protein-misfolding diseases

Despite the apparent overall preponderance of HSP70-HSP40 system components in our survey, one additional family was prominent, observed in 19% of the studies. Out of the 19 chaperome members that were identified five or more times, 32% are members of the ATP-independent sHSP family. sHSPs form large, dynamic oligomers that bind partially unfolded proteins, functioning without the use of ATP to drive substrate binding and release (Bakthisaran et al., 2015). Overall, sHSPs act as holdases, providing a shield to prevent aberrant protein-protein interactions (Fig. 4) and there are at least 10 sHSP family members in humans. Our summary (Table 1) points towards specific sHSPs that play a prominent role in misfolding diseases, as judged by frequency of observations, including CRYAB, HSPB1, HSPB3 and HSPB8 (each 7×), HSPB6 (6×), and CRYAA (5×) (Fig. 1).

Although the preponderance of members of the HSP70-HSP40 system is not surprising, the frequency at which sHSPs were identified supports a role of sHSPs in disease progression that has not been often explored. The importance of sHSPs in disease was originally noted from the observations that HSPB1 and CRYAB were overexpressed in AD brains (Shinohara et al., 1993; Renkawek et al., 1994a,b) and HSPB1, CRYAB, HSPB6 and HSPB8 were associated with AD plaques (Shao et al., 2012). Furthermore, sHSPs were found to be consistently upregulated in the aging human brain and in the context of neurodegenerative diseases (Brehme et al., 2014). Activation of sHSPs could be indicative of a more general stress response mechanism to provide neuroprotection in aging. A recent proteome-wide study in *C. elegans* with extended lifespan found that sHSPs associate with misfolded protein aggregates (Walther et al., 2015). sHSPs might shield or sequester misfolded proteins, delaying proteostasis decline during aging. In support of this, our survey highlights specific sHSP family members as relevant for misfolding diseases as judged by frequency of observations in a diverse range of studies (Fig. 1).

## Underrepresented or missing chaperones and co-chaperones

Our collective analysis of unbiased approaches in model systems of protein-misfolding disease points towards prominent individuals and families within the chaperome that are crucial for proteostasis. Based on the frequency of identification in a representative set of studies, we suggest that the overrepresented chaperome families and members play a key role in protein-misfolding diseases. In addition, this survey also uncovered chaperome families that were not identified as often as anticipated (Table 1). Surprisingly, HSP90 (HSP90AA1 and HSP90AB1) and HSP60 (CCT/TRiC) were collectively found by relatively few studies, i.e. four or fewer studies each (Fig. 1). Furthermore, it is striking that out of the wide variety of TPR-domain proteins that function as co-chaperones to HSP70 and HSP90 family members (Prodromou et al., 1999; Song and Masison, 2005), only a few were identified, most of which were identified in a few studies or only once (Fig. 1). Although these chaperome families did not emerge from our survey of genetic approaches in model systems, studies not discussed in this review have demonstrated the importance of the functionality of HSP90s, HSP60s and TPR-domain proteins in diseases of protein misfolding (Behrends et al., 2006; Kitamura et al., 2006; Tam et al., 2006; Taipale et al., 2010; Al-Ramahi et al., 2006; Wolfe et al., 2013).

There are many reasons as to why specific candidates or families might appear at a lower frequency or not at all. Certain chaperones or co-chaperones might have specialized rather than general roles in proteostasis, recognizing certain low-abundance substrates or cell type-specific substrates. Although misfolding of these low-level candidates could generate imbalances in proteostasis, the effect sizes might be small, reducing the frequency of observation. Furthermore, the selected studies examined protein misfolding within the cytosol; however, underrepresented chaperones and co-chaperones could function within specific subcellular compartments. In a handful of cases, we did identify ER- and mitochondria-specific chaperones and co-chaperones (Figs 1 and 2), but at a lower frequency. In addition to specialized functionality, chaperones and co-chaperones do not work in isolation but rather within a network of chaperone machines that is itself embedded inside the PN. Therefore, compensatory networks could functionally overcome the deficit of an individual chaperone, masking its effect on proteostasis. Finally, studies where the level of knockdown or overexpression of the clone was insufficient to generate detectable changes in toxicity or aggregation can limit our ability to accurately assess the function of ‘missing’ candidates. Despite these challenges, we propose that the combined strength of multiple unbiased studies using model systems of protein misfolding can inform strategies for forward progress, as outlined below.

## Model systems as a guide for therapeutic chaperone-targeting strategies

Model systems have led the way in demonstrating the importance of chaperone function in diseases of protein misfolding (Brodsky, 2014). Deficiencies in the ability of chaperones to maintain proteostasis lead to perturbations in the folding, trafficking and clearance of specific disease proteins (Broadley and Hartl, 2009; Valastyan and Lindquist, 2014). Therapeutic approaches to overcome proteostasis deficiencies have largely focused on the activation of HSF1, the heat shock transcription factor responsible for simultaneous upregulation of the expression of multiple molecular chaperones during stress (Calamini et al., 2011; Pierce et al., 2013). Given the level of connectivity and combinatorial complexity underlying systems-level modulation of the chaperome, therapies that target the chaperome globally in protein folding and proteostasis disorders could generate undesirable side effects by changing the overall cellular folding landscape. Findings provided by genetic studies summarized in this review can channel our efforts in developing targeted therapeutics to minimize off-target effects while preventing, delaying and remedying disease pathology. Based on the compilation of genes presented in Table 1, potential therapeutic strategies include increasing the levels and functionality of both the HSP70-HSP40 system and sHSP family members.

Based on the prevalence of HSP70 family members identified in our survey, a promising strategy could center on the development of novel (isoform-specific) targeted modulators of HSP70 activity. Multiple studies in model systems demonstrate that overexpression of HSP70 can reduce toxicity and protein aggregation. HSP70 family members are abundant, highly conserved and play a central role in proteostasis maintenance (Fig. 4) (Mayer and Bukau, 2005). Therefore, a disease-related protein is likely to encounter HSP70 family members at multiple points throughout its lifetime, implying that HSP70 could be a suitable target for therapeutic intervention in a broad spectrum of protein-misfolding diseases. However, the biogenesis of cellular proteins might be affected by general modulation of HSP70 levels, possibly leading to undesired cytotoxic effects. Given its central role within the PN, modulators of this family must be designed to adequately and specifically target relevant HSP70 members to minimize overall perturbations of the cellular proteome (Fig. 3). Compounds such as the allosteric inhibitor of ATP-binding for the inducible HSP70 isoform HSPA1A/HSPA1B, called HS-72, will likely lead to beneficial consequences (Howe et al., 2014). Alternatively, chaperones can be modified for enhanced activity against disease substrates, while preserving their functional specificity towards canonical substrates (Aprile et al., 2015). Therapeutically, this is likely to be more challenging. A systems-level understanding of the wiring of the chaperome and its interface with the PN will enable us to move from a global assault to targeted intervention on specific proteostasis regulators, fine-tuning specific functional arms within the PN.

Shifting efforts from targeting global changes in HSP70 level and activity towards designing HSP70 modulators with specific effects are underway (Wisen et al., 2010; Cesa et al., 2013; Brandvold and Morimoto, 2015). As described above, the HSP70 chaperone machine is modulated by co-chaperones such as HSP40s, with distinct combinations of HSP70s and HSP40s facilitating folding, transport, refolding, preventing aggregation, or promoting disaggregation and degradation (Fig. 3). Recent therapeutic strategies have focused on partitioning HSP70 activity towards prevention of aggregation, disaggregation and degradation. To redirect HSP70 activity, altering interactions of HSP70s with specific co-chaperones might selectively enhance or inhibit function. Small molecules are being designed that could either disrupt or promote interactions of HSP70s with HSP40s (Wisen et al., 2010; Cassel et al., 2012; Cesa et al., 2013). With the large number of HSP70 and HSP40 family members, one challenge that researchers face is that finding the relevant, client-specific, functional combination of HSP40 and HSP70 could increase complexity. However, efforts on specific combinations can be guided by information revealed by surveying the wealth of information from interactome-mapping or genetic studies using model systems (Taipale et al., 2014) (Table 1). The HSP40 proteins DNAJB4 and DNAJB6 play a prominent role in modulating proteostasis in the presence of polyQ disease proteins. Identifying modulators of these particular combinations could regulate HSP70 activity in a direction that most effectively ameliorates disease pathology.

In addition to the HSP70-HSP40 system, sHSPs were identified frequently in multiple models expressing diverse disease proteins. As sHSPs have been shown to interact and sequester aggregation-prone proteins, increasing the levels of sHSPs could provide protection as cells accumulate misfolded proteins during aging (Fig. 4). Small molecules that activate the heat-shock response will lead to an increase in sHSP levels. Given that sHSPs are highly conserved and have pleotropic functions, a general increase in their activity will likely have side effects. Therefore, developing therapeutics that can specifically activate subsets of sHSPs could prime a cell for protection from particular misfolded disease proteins. Using the collective data generated from model systems, targeting an increase in CRYAB, HSPB1, HSPB3, HSPB6, and HSPB8 could be a reasonable strategy to follow (Fig. 1). Alternatively, rational design of biological drugs that mimic sHSP or specific chaperone domain interfaces with disease targets, such as sHSP-derived peptides with chaperone-like activities (mini-chaperones) or Brichos domains that can inhibit formation of neurotoxic oligomers of Aβ42, could represent a powerful therapeutic strategy for protein-misfolding diseases (Raju et al., 2012; Cohen et al., 2015).

## Conclusions and future outlook

Identifying key chaperones that are compromised in disease is central to designing novel therapeutic approaches for proteostasis regulation in protein-misfolding diseases. Our survey of research articles using a variety of approaches in model systems suggests that a specific subset of molecular chaperones, the HSP70-HSP40 system and small HSPs in particular, play a fundamental role across protein-misfolding diseases. We also highlight novel, lesser-studied chaperones, including TPR co-chaperones not previously implicated in proteostasis control, aging or disease. The information compiled from this survey could guide efforts in the development of small molecules to treat protein-misfolding diseases. Due to the central role of molecular chaperones in proteostasis, toxic side effects remain a major concern when modulating the activity of a conserved ubiquitous target. Careful adjustment of chaperone activity should allow targeted alleviation of folding deficiencies while limiting side effects on other client proteins with no disease context. Small-molecule screens using model systems expressing disease proteins have been performed to identify suppressors of aggregation and restoration of cellular function (Calamini et al., 2011). We propose that model systems expressing disease proteins can be used to not only evaluate the effectiveness of small molecules on protein aggregation and cellular toxicity but also to test the effects of small molecule treatments. Model systems treated with small molecules can be scored for a variety of phenotypic readouts that reflect overall health such as fecundity, healthspan and lifespan, followed by the synthesis and testing of chemical analogs that increase potency and specificity, and reduce toxicity.

An increasing understanding of the chemical biology of molecular chaperones, systems-level functional maps of chaperome connectivity, chaperome dynamics, and their role in proteostasis maintenance provides a vast target space for therapeutic modulation in disease intervention (Brandvold and Morimoto, 2015). The chaperome lies at the center of the PN. As our survey of genetic studies using model organisms expressing misfolded proteins identified key chaperones that modulate proteotoxicity (Table 1), a similar approach can be extended to the entire PN. Analysis of frequency of observations of PN components can point towards therapeutic targets for designing novel drugs that regulate the PN. Subsequently, model systems provide a tool to optimize effectiveness and reduce toxicity of these new small molecules. Based on the importance of proteostasis in health and disease, we suggest that chaperome- and PN-targeted therapeutic interventions could be beneficial for a large number of protein-misfolding diseases including age-related neurodegenerative disorders. We anticipate that model systems will continue to provide powerful tools to guide therapeutic discovery, while helping to reduce toxic side effects through fine-tuned proteostasis regulation.
